# mTOR Signaling in Cardiometabolic Disease, Cancer, and Aging

**DOI:** 10.1155/2017/6018675

**Published:** 2017-07-10

**Authors:** Anindita Das, Flávio Reis, Yasuhiro Maejima, Zhiyou Cai, Jun Ren

**Affiliations:** ^1^Pauley Heart Center, Virginia Commonwealth University, Richmond, VA 23298, USA; ^2^Laboratory of Pharmacology & Experimental Therapeutics, Institute for Biomedical Imaging and Life Sciences (IBILI), Faculty of Medicine, University of Coimbra, 3000-548 Coimbra, Portugal; ^3^Centre for Neuroscience and Cell Biology-Institute for Biomedical Imaging and Life Sciences (CNC.IBILI) Research Consortium, University of Coimbra, 3004-517 Coimbra, Portugal; ^4^Department of Cardiovascular Medicine, Tokyo Medical and Dental University, 1-5-45 Yushima, Bunkyo-ku, Tokyo 1138519, Japan; ^5^Department of Cell Biology and Molecular Medicine, Cardiovascular Research Institute, Rutgers New Jersey Medical School, 185 South Orange Ave, MSB G-609, Newark, NJ 07103, USA; ^6^Shiyan Renmin Hospital, Hubei University of Medicine, Hubei, China; ^7^College of Health Sciences, University of Wyoming, Laramie, WY, USA

The mammalian target of rapamycin (mTOR), an evolutionarily conserved serine/threonine kinase, plays a significant role in integrating cellular and environmental cues that modulate cell metabolism, growth, proliferation, survival, and homeostasis. The distinct roles of mTOR were identified in gene transcription, protein synthesis, tissue regeneration and repair, oxidative stress, immunity, aging, and cell death that include autophagy and apoptosis (Figure 1(a)). Emerging evidence over the last decade indicates that deregulation of mTOR signaling has been implicated in many human diseases, including cancer, obesity, diabetic complications, pulmonary hypertension, cardiovascular diseases, and neurodegeneration (Figure 1(b)). Moreover, oxidative stress has also been etiologically implicated in this wide variety of disease processes and states. Notably, the mTOR pathway is activated during various cellular processes including tumor formation and angiogenesis, insulin resistance, adipogenesis, and T-lymphocyte activation. Based on its pathophysiological importance, the mTOR signaling pathway has attracted broad scientific and clinical interest as a potential therapeutic target to treat a variety of diseases associated with oxidative stress, aging, proliferative disorders, and metabolic abnormalities. The focus of this special issue is to advance our understanding of the mTOR signaling pathways in metabolic and cardiovascular diseases as well as cancer and aging, which could be pivotal for the development of novel therapeutic strategies to treat many human diseases. Internationally recognized experts highlight the distinct role of mTOR signaling in cardiovascular and metabolic diseases as well as cancer with insightful presentation to enrich our knowledge in emerging therapeutic application of mTOR inhibitors.

Acute myocardial infarction (AMI) is a leading cause of death worldwide. Reperfusion of the coronary flow is obligatory to resuscitate the ischemic myocardium; however, it may cause a paradoxical cardiomyocyte dysfunction, which is known as “reperfusion injury.” There is still no effective therapy for preventing myocardial reperfusion injury. Using the left coronary artery occlusion model in C57 mice, S. M. Filippone et al. revealed that rapamycin administration at the onset of reperfusion attenuates myocardial infarction. Rapamycin improves post-MI/R (myocardial ischemia/reperfusion) cardiac function with reduction of oxidative stress and cardiomyocyte apoptosis. Considering the important role of Reperfusion Injury Salvage Kinase (RISK) pathway (specifically AKT and ERK1/2), they further elucidated the effects of rapamycin on mTOR complexes (mTORC1 and mTORC2) and MAP kinase (ERK and p38) signaling pathways. This study confirmed that reperfusion therapy with rapamycin protects the heart against MI/R injury by selective activation of mTORC2 (indicated by the induction of phosphorylation of AKT S473) and ERK with simultaneous inhibition of mTORC1 (indicated by the attenuation of phosphorylation of ribosomal protein S6) and p38.

Atherosclerotic plaque instability and rupture is the major cause of acute myocardial infarction and stroke. The vascular smooth muscle cell (VSMC) is a major contributor to atherosclerotic plaque progression and vulnerability. The imbalance of autophagy of VSMC in the fibrous cap might cause plaque instability. mTOR is a key regulator of autophagy which may be involved in the development of atherosclerotic plaque. Using murine atherosclerosis model, Z. Luo et al. revealed that chronic treatment with low dose (50 mg/kg/d for 16 weeks) rapamycin (a specific mTOR inhibitor) alleviates atherosclerosis progression and promotes plaque stability by moderately inducing autophagy of VSMC. They demonstrated that moderate autophagy induced by low dose rapamycin was able to attenuate VSMCs senescence. Inhibition of mTORC1 by rapamycin repressed ULK1 which led to the formation of an active ATG1-ATG13-ATG17 complex and stimulation of autophagy. The p53 family plays a pivotal role in cellular senescence and the inhibition of this pathway prevents the development of atherosclerosis by effectively suppressing proinflammatory cytokines, as well as cellular senescence. In this study, they also exhibited that mTORC1 inactivation with rapamycin also inhibited VSMCs senescence by repressing the expression of p53. Therefore, rapamycin-induced vascular cell autophagy may provide novel insights into the clinical treatment of atherosclerosis.

Sonodynamic therapy (SDT), a novel noninvasive approach, exhibited a beneficial effect on atherosclerosis by inducing autophagy and apoptosis of macrophages with stabilizing the atherosclerotic plaques. Y. Jiang et al. investigated whether the combination of hydroxysafflor yellow A (the hydrophilic fraction of the safflower plant) and ultrasound (HSYA-SDT) could induce autophagy and inhibit inflammation in THP-1 macrophages. They showed that autophagy was induced by HSYA-SDT in human THP-1 macrophages with inhibition of inflammatory factors. Based on their results, they suggested that HSYA-SDT induces an autophagic response via the PI3K/AKT/mTOR signaling pathway and inhibits inflammation by reactive oxygen species in THP-1 macrophages.

The persistent hyperactivation of mTOR has been implicated in diverse metabolic disorder, including obesity, type 2 diabetes, and metabolic syndrome. Diabetic patients are more prone to cardiovascular diseases which is associated with worse prognosis as compared to its nondiabetic counterpart. The beneficial effect of rapamycin treatment in diabetic mice was well documented. Rapamycin improves metabolic status by regulating glucose metabolic proteins and prevents cardiac dysfunction in type 2 diabetic mice by attenuating oxidative stress and modulating antioxidant and contractile proteins. Rapamycin also protects diabetic heart against reperfusion injury through STAT3 signaling. Inhibition of mTORC1 with rapamycin reduces body fat and lean mass in male Zucker obese (ZO) rat. Rapamycin treatment for 12 weeks also increased expression of cardiac Mediator Complex 13 (MED13) which is known to improve whole body metabolism. In this special issue, C. Luck et al. for the first time revealed that long time treatment with rapamycin modulates several intracardiac proteins differentially in healthy Zucker lean rats and diabetic Zucker obese rats. They showed that rapamycin treatment causes an increase in cardiac fibrosis in healthy Zucker lean rats, whereas rapamycin suppresses cardiac fibrosis in Zucker obese rats by differentially regulating the phosphorylation of AKT and antifibrotic cytokines in healthy and obese rats. Moreover, rapamycin treatment for six weeks improves many of the cardiac parameters in both healthy and obese/diabetic rats. However, continuation of the treatment for another six weeks reversed these cardiac functional improvements in Zucker obese rats. Interestingly, they reported that long term treatment with rapamycin enhances the fasting glucose level in Zucker obese rats with activation of nonoxidative glucose pathways (NOGPs), particularly the advanced glycation end products (AGE) pathway. Understanding the differential effects of rapamycin on cardiac tissues in diabetic rats compared to healthy subjects is an imperative aspect to ensure the efficient application of this drug for cardioprotection in patients with preexisting diabetes.

X. Han et al. explored the role of acarbose, an oral glucose-lowering drug, on diabetes-related wound healing, angiogenesis, and endothelial precursor cell (EPC) impairment, focusing on the AKT/eNOS (endothelial nitric oxide synthase) signaling pathway. The authors suggested that acarbose has beneficial effects on diabetes-induced wound healing and angiogenesis by improving EPC function, using the db/db mice as animal model of type 2 diabetes mellitus and* in vitro* functional assays with isolated bone marrow EPCs (BM-EPCs). They showed enhanced AKT and eNOS activation in cultured BM-EPCs from acarbose-treated db/db mice, together with increased nitric oxide production. Furthermore, AKT inhibition was able to abolish the beneficial effect of acarbose on high glucose induced EPC dysfunction* in vitro*, which was accompanied by reduced eNOS activation. Overall, the authors concluded that acarbose treatment could reverse the diabetes-related impairment of wound healing, angiogenesis, and EPC function by activating the AKT/eNOS signaling pathway.

Finally, the review article contributed by D. Zhao and coworkers discussed the role of oxidative stress and mTOR signaling in myocardial ischemia/reperfusion (MI/R) injury under diabetes. They summarized the potential mechanisms underlying diabetic heart damage when subjected to MI/R injury, including impaired activation of prosurvival pathways, excessive endoplasmic reticulum (ER) stress, increased basal oxidative state and decreased antioxidant defences, impaired autophagy and mitochondrial quality control, and mTOR overactivation. The authors described the controversial data regarding the cardioprotective or cardiotoxic role of the mTOR signalling in MI/R injury and the complex interplay with oxidative stress. Finally, potential therapeutic strategies against cardiac injury in diabetic patients were discussed, including the use of the hypoglycemic agent metformin and the newly found free radicals scavengers Sirt1 and CTRP9, which might be viewed as promising pharmacological cardiometabolic targeted therapeutic genes to protect the diabetic heart from MI/R injury.

Aberrant activation of the mTOR signaling is involved in some cancer hallmarks, suggesting mTOR as an attractive target for cancer therapy. In the review article by F. Palavra et al., the authors described the recent advances and the challenges of mTOR inhibitors use in the treatment of patients with tuberous sclerosis complex (TSC), an autosomal dominant genetic disease associated with mutations in the TSC1 and TSC2 genes, leading to the overactivation of mTOR signalling pathway. TSC is a disorder of cellular differentiation and proliferation, characterized by the presence of benign and noninvasive tumor-like lesions (called hamartomas) that can affect multiple organ systems, such as the brain, kidney, skin, heart, lung, and liver. The authors firstly summarized the clinical diagnostic criteria for TSC and the molecular upstream and downstream mediators of mTOR-TSC regulation; afterwards, they revisited the pharmacological aspects of mTOR inhibitors (rapamycin, also known as sirolimus, and its analogues everolimus, temsirolimus, and ridaforolimus), focusing on the clinical trials leading to their approval in TSC-related conditions. To conclude, authors exposed the current challenges and future directions in TSC treatment, including the possibility of using the second generation of mTOR inhibitors (known as mTOR kinase inhibitors or TORKinibs), which allow inhibition of both mTORC1 and mTORC2, thus opening new windows of opportunity to better treat TSC and related conditions.

The review manuscript by A. C. de Melo et al. introduced the recent progress regarding the mTOR pathway inhibitors for the treatment of gynecological malignancies, including endometrial, cervical, and ovarian cancers. As the efficacy of the currently available antitumor agents is still limited, the prognosis of the advanced gynecologic cancers remains poor. Therefore, more effective therapeutic approaches for gynecological malignancies are desperately needed. In this regard, PI3K-AKT-mTOR-mediated signaling would be one of the most promising therapeutic targets in gynecologic cancers because aberrant PI3K-dependent signaling occurs oftentimes in various types of cancer, including endometrial, cervical, and ovarian cancers. Initially, the authors summarized the clinical characteristics, involvement of PI3K-AKT-mTOR-mediated signaling, and the recent advances of clinical trials using mTOR pathway inhibitors in endometrial, cervical, and ovarian cancers, respectively. Next, they illustrated the effects of mTOR inhibitors in both the case of monotherapy and those of combination therapy with other cytotoxic drugs against gynecologic cancers. Then, they concluded that more intensive exploration of PI3K-AKT-mTOR-targeted drugs which can get over the advanced or recurrent gynecologic cancers should be conducted.

In the review article of S. Faes et al., the mechanisms underlying resistance to mTORC1 inhibitors in cancer therapy were revisited, including the existence of mTOR mutations, the activation of alternate proliferative signaling pathways following mTORC1 inhibition, and the intratumoral heterogeneity of mTORC1 activity. The molecular mechanisms associated with those conditions were dissected, including the main mutations already identified, namely, in the FRB domain (FKBP-rapamycin binding domain) of mTOR, which hamper the anticancer efficacy of rapalogs by blocking the binding of rapamycin-FKBP12 to mTOR, as well as mutations that promote hyperactivation of mTOR and resistance to ATP-competitive mTOR inhibitors. Furthermore, the authors discussed the alternate proliferation pathways that have been described, such as the overactivation of PI3K/AKT and RAS/RAF/MEK/MAPK, secondary to the inhibition of negative feedback loops after mTORC1 inhibition. Finally, the key elements contributing to the intratumoral heterogeneity, including genetic and functional heterogeneity, as well as tumor hypoxia and pH environment, were discussed. It was concluded that those mechanisms, in particular tumor heterogeneity, should be considered when developing new therapeutic anticancer approaches targeting mTOR.

Ultraviolet (UV) radiation is the most environmental skin aggressor, causing skin disorders such as erythema or sunburn, premature skin aging, and skin cancer. UVB-induced skin damage is caused by DNA damage, apoptosis, ER stress, and activation of key signaling pathways (e.g., MAPK family and AMPK). UVB was reported to increase phosphorylation of mTOR substrate 4E-BP1 and p70 S6 kinase in epidermal keratinocytes. Keratinocytes, the predominant component in the epidermis, are the major target of UVB induced skin damage. S. Xu et al. detected the sensitivity of human keratinocyte cell line HaCaT and human epidermal keratinocytes (HEKs) to four different inhibitors of mTOR, rapamycin, everolimus, Torin 1, or pp242. They reported that keratinocytes were sensitive to those mTOR inhibitors, but the regulation of mTOR downstream signaling was distinct. Only rapamycin induces autophagy in HaCaT cells among the four mTOR inhibitors tested. Therefore, they indicated that mTOR inhibition in keratinocytes cannot always induce autophagy. Moreover, they exhibited that mTOR signaling is insensitive to UVB but sensitive to UVA radiation. The mTOR inhibition caused by rapamycin, everolimus, or pp242 does not interfere UVB-stimulated the series of biological events in keratinocytes, including the downregulation of the ER molecular chaperone BIP and ER transmembrane protein PERK, activation of the DNA damage marker Histone H2A and stress activated protein kinase SAPK/JNK, and cleavage of apoptotic molecular caspase 3 and PARP. Accordingly they suggested that mTOR signaling does not play a crucial role in the complex cellular responses in keratinocytes with ultraviolet damage.

## Figures and Tables

**Figure 1:**
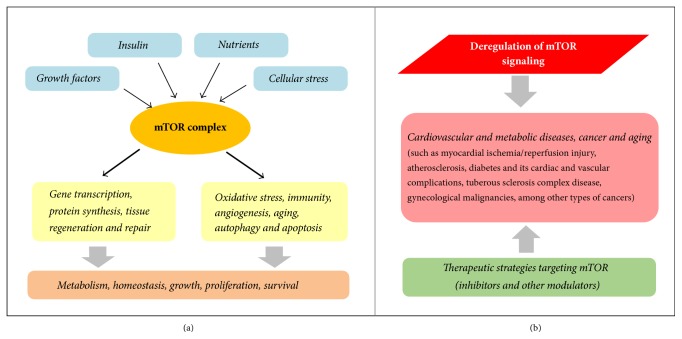
Schematic representation of the mTOR signaling network (a) and the involvement of deregulated mTOR signaling in cardiovascular and metabolic disorders, in cancer and in aging (b), such as those highlighted in this special issue: myocardial ischemia/reperfusion injury, atherosclerosis, diabetes and its cardiac and vascular complications, tuberous sclerosis complex disease, and gynecological malignancies, among other types of cancers.

